# Prevalence and mortality of epilepsies with convulsive and non-convulsive seizures in Kilifi, Kenya

**DOI:** 10.1016/j.seizure.2021.04.028

**Published:** 2021-05-14

**Authors:** Symon M Kariuki, Anthony K Ngugi, Martha Z Kombe, Michael Kazungu, Eddie Chengo, Rachael Odhiambo, Amek Nyaguara, Brian G Neville, Charles RJC Newton

**Affiliations:** a KEMRI/Wellcome Trust Research Programme, Kilifi, Kenya; b Studies of Epidemiology of Epilepsy in Demographic Surveillance Systems (SEEDS) – INDEPTH Network, Accra, Ghana; c Department of Public Health, Pwani University, Kilifi, Kenya; d Department of Psychiatry, University of Oxford, Oxford, United Kingdom; e Department of Population Health, Medical College (East Africa), Aga Khan University, Nairobi, Kenya; f Foundation for People with Epilepsy, Malindi, Kenya; g Neurosciences Unit, UCL Institute of Child Health, London, United Kingdom

**Keywords:** Epilepsy, Non-convulsive seizures, Convulsive seizures, Mortality, Prevalence, Low- and middle-income countries

## Abstract

**Objectives:**

The prevalence of all epilepsies (both convulsive and non-convulsive seizures) in Low- and Middle-Income Countries (LMIC), particularly sub-Saharan Africa is unknown. Under estimation of non-convulsive epilepsies in data from these countries may lead to inadequate and sub-optimal allocation of resources to control and prevent epilepsy. We determined the prevalence of all types of epilepsies and compared the mortality between convulsive seizures and non-convulsive seizures in a resource limited rural area in Kenya.

**Methods:**

Trained clinicians identified cases of epilepsy in a randomly selected sample of 4,441 residents in the Kilifi Health and Demographic Surveillance System site using a cross-sectional survey design. Seizure types were classified by epileptologists using the current guidelines of the International League Against Epilepsy (ILAE). We estimated prevalence for epilepsy with convulsive seizures and non-convulsive seizures and for epilepsy with non-convulsive seizures only and compared premature mortality between these groups of seizures.

**Results:**

Of the 4441 people visited, 141 had lifetime epilepsy and 96 active epilepsy, which is a crude prevalence of 31.7/1,000 persons (95% CI: 26.6-36.9) and 21.6/1,000 (95% CI: 17.3-25.9), respectively. Both convulsive and non-convulsive seizures occurred in 7% people with epilepsy (PWE), only convulsive seizures in 52% and only non-convulsive seizures in 35% PWE; there was insufficient information to classify epilepsy in the remainder 6%. The age- and sex-adjusted prevalence of lifetime people was 23.5/1,000 (95% CI: 11.0-36.0), with the adjusted prevalence of epilepsy with non-convulsive seizures only estimated at 8.2/1,000 (95%CI:3.9-12.6). The mortality rate in PWE was 6.3/1,000 (95%CI: 3.4-11.8), compared to 2.8/1,000 (2.3-3.3) in those without epilepsy; hazard ratio (HR) =2.31 (1.22-4.39; p=0.011). The annual mortality rate was 11.2/1,000 (95%CI: 5.3-23.4) in PWE with convulsive and non-convulsive seizures and none died in PWE with non-convulsive seizures alone.

**Conclusions:**

Our study shows that epilepsy with non-convulsive seizures is common and adds to the prevalence of previously reported estimates of active convulsive epilepsy. Both epilepsy with convulsive seizures and that with non-convulsive seizures should be identified for optimising treatment and for planning resource allocation.

## 1 Introduction

Epilepsy is one of the most common chronic neurological disorders and is an important public health problem affecting close to 70 million people worldwide [1]. This number approximates about 0.7% of the global burden of disease and contributes up to 17 million disability adjusted life-years (DALYs) annually [2]. Up to 90% of the people with epilepsy (PWE) reside in low and middle income countries (LMICs) [1], with 20% of the global burden in Africa alone [3]. Another 500 million people are affected indirectly as parents, relatives and friends [4]. Proximate causes, features and consequences of epilepsy with convulsive seizures have been described [5], but these have not been systematically studied for epilepsy with non-convulsive seizures in LMIC.

Most epidemiological studies in Africa have focused on epilepsy with convulsive seizures, since these seizures are easier to detect in cross-sectional surveys and are thought to be associated with more morbidity and premature mortality [6]. Non-convulsive seizures are more difficult to detect, and often need experienced clinicians to elicit the subtle manifestations. Two previous studies have estimated crude prevalence of epilepsy with convulsive and of epilepsy with non-convulsive seizures at 10.2/1,000 and 12.5/1,000, in Kenya and Zambia respectively [7,8]. Neither study used experienced clinicians to screen the populations, and both noted the limitations of their studies to identify non-convulsive seizures. Furthermore, neither study estimated the prevalence of life-time epilepsy.

Cross-sectional studies are thought to underestimate the prevalence of life-time epilepsy since non-convulsive seizures are difficult to detect and may constitute up to 50% of all epilepsies in population-based studies [9]. This preponderance of studies of epilepsies with convulsive seizures is primarily due to scarcity of resources and manpower for detecting non-convulsive seizures, which may occur in 17-50% of all epilepsies [9,10]. The paucity of epidemiological data on epilepsy with non-convulsive seizures underestimates the true burden of epilepsy in LMIC, complicates comparison of estimates with those from the high-income countries (HIC) and affects planning and allocation of resources for epilepsy care.

A number of cross-sectional surveys to determine the prevalence of active convulsive epilepsy have been conducted in the Kilifi Health and Demographic Surveillance System (KHDSS) study area in Kenya (http://www.kemri-wellcome.org/khdss/). Two small surveys estimated prevalence at 4.0/1,000 using a combination of one round survey and key-informants [11] and 11/1,000 in children aged 6–9 years using a two round survey [12]. A more recent three-stage prevalence survey estimated the prevalence of active convulsive-epilepsy at 7.8/1,000 after adjusting for attrition-between stages and sensitivity of the screening methodology [13].

We conducted a cross-sectional survey to estimate the prevalence of epilepsy with convulsive seizures and that with non-convulsive seizures in a randomly selected sample of the KHDSS population in in which the population was screened by clinical officers who had been trained in the diagnosis of epilepsy. We further classified the seizure types that characterize the identified cases and followed-up the sample to assess premature mortality.

## 2 Methods and materials

### 2.1 Study setting

We conducted the study within the KHDSS (http://www.kemri-wellcome.org/khdss/) which covers an area of 891 km^2^. It is located in Kilifi District, one of the poorest districts in Kenya comprising of a population of 233,880 persons in 2008 living in 25,526 homesteads [14]. The KHDSS area was mapped using global positioning system (GPS) receivers, and digital maps derived from these data were used to locate homesteads and follow up study participants. Community based field workers conduct re-enumeration and vital status registration to update the population registers by visiting every homestead two or three times per year. The population is served by the Kilifi County Hospital, which is located at the centre of the KHDSS area. The main causes of paediatric admissions to hospital are neonatal insults, infections of the brain and head injury [13,15], which may be important risk factors for epilepsy in this area.

Residents of the study area are Mijikenda, which is a Bantu grouping of nine tribes with Girima (45%), Chonyi (33%) and Kauma (11%) being the most common. About 55% of the population is considered absolutely poor with per capita monthly income of about 10 USD. The majority (80%) depend on subsistence farming, which is constrained by the low agricultural potential of the land (only 19% is arable). Only 45% of people are literate. During the years of the study, crude birth and death rates for this area are approximately 35/1,000 and 6/1,000 per year respectively, and migration rates are approximately 100/1,000 per year, most of which is within the study area [14].

### 2.2 Study population and cross-sectional survey

Using a prevalence estimate of 4.5/1,000 [13,16] we estimated that a minimum sample size of 3,263 people was required to determine the prevalence of all types of epilepsies in Kilifi [17,18]. To account for loss to follow-up and meet the sample size requirements for an embedded validation study [19], a sample of 5,796 people resident in the study area was randomly selected from the 2008 KHDSS census database for this study.

**Fig. 1:**
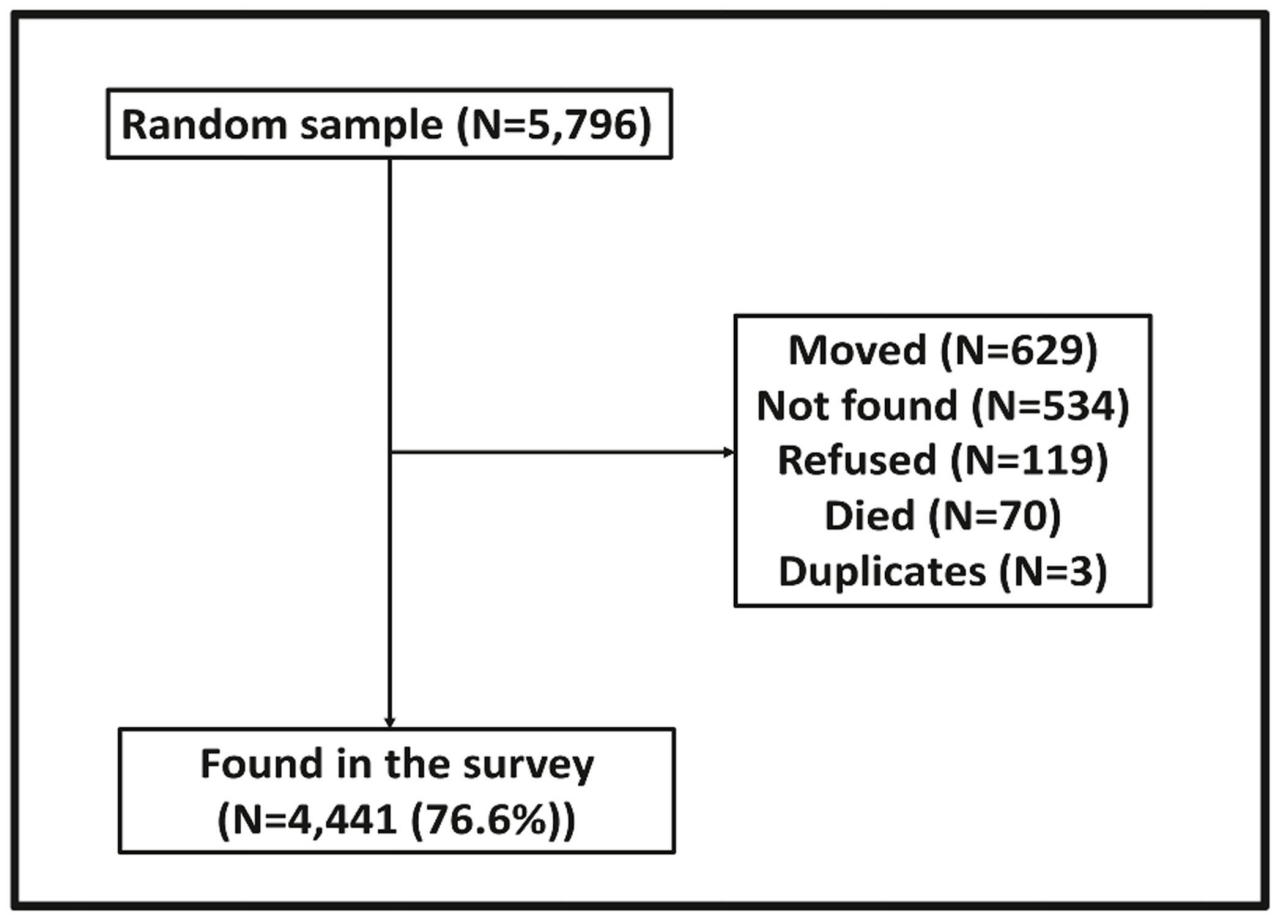
Follow-up of participants in the cross-sectional survey

The study clinicians (experienced in the diagnosis and management of epilepsy) visited the homes of 5,796 people randomly chosen from the KHDSS database, to recruit them into the study. The clinicians performed a detailed clinical history to identify PWE among those found at home (numbers shown in Fig. 1). Epilepsy was defined as two or more unprovoked seizures in a life-time where least 2 of the seizures occurred ≥ 24 hours apart [20,21]. After the field survey, all case notes were reviewed independently by two neurologists (CRN and BN) to confirm the diagnoses assigned by the study clinicians. Psychogenic non-epileptic seizures and syncope were excluded on the clinical history, since further investigations such as electrocardiogram (ECG) and vid-eo/EEG telemetry were not possible in the field. Psychogenic non-epileptic seizures were suspected if the person resisted attempts to restraint limb movement, forcefully closed eyes during the entire seizure, or had an event only in public, and never when alone. Syncope was suspected if, for example, the seizures were due to prolonged standing in hot crowded places, or if there was rapid recovery from the seizure without post-ictal confusion or drowsiness.

The two neurologists also classified seizure types from available data, using the recommendations of the Commission of the Classification and Terminology of the ILAE. The seizure semiology information was used to categorize epilepsy into: (i) epilepsy with convulsive seizures, which included non-convulsive seizures; and (ii) epilepsy with non-convulsive seizures, which excluded convulsive seizures. This study was conducted during the period 2^nd^ May 2009 to 30^th^ April 2011, and the people with epilepsy were followed four-monthly for mortality status until 31^st^ December 2020.

### 2.3 Analysis

All data were double entered and verified in MySQL Version 5 open-source database (Oracle Corporation, Redwood Shores, CA, USA). Prevalence of all epilepsies was estimated as the number confirmed to have epilepsy divided by the number interviewed and expressed per 1000 persons with 95% confidence intervals (CI). Prevalence was estimated by age and sex and for epilepsies with convulsive seizures plus non-convulsive seizures and for those with non-convulsive seizures alone. Exploratory comparison of frequencies was done with Pearson’s Chi-squared test (X^2^) or Fisher’s exact test (when observations were infrequent). Mortality rate was computed as a function of number of deaths divided by person years of observation. All analyses were performed in STATA version 15 (StataCorp, College Station, TX, USA).

### 2.4 Ethical considerations

Written informed consent was obtained from all study participants. Approval for the study was obtained from the Kenya Medical Research Institute/ National Ethical Review Committee.

## 3 Results

### 3.1 Follow-up of participants

Out of the random sample of 5,796 participants, 1355 (23.4%) were could not be identified: 629 (46.4%) had moved, 534 (39.4%) could not be traced, 119 (8.8%) refused consent and 70 (5.2%) had died, while 3 (0.2%) were found to have been duplicate records (Fig. 1). Among the 4,441 found (Table 1), 53.5% were females. The attrition from the cohort did not vary by age (P=0.1) or sex (P=0.9).

### 3.2 Prevalence of life-time epilepsy and active epilepsy

A total of 141 people reported a history of at least 2 unprovoked seizures yielding a crude life-time prevalence of 31.7/1,000 persons (95% CI: 26.6-36.9). The prevalence of lifetime epilepsy was 31.6/1,000 (95% CI: 24.5-38.6) in females and was 32.0 (95% CI: 24.4-39.6) in males, being similar between the sexes (P=0.936). The prevalence of lifetime epilepsy did not vary significantly by age (P=0.136), although it was lowest in the youngest (0-5 year) age group (21.6 per 1,000 (95%CI: 12.0-31.2)) and highest in oldest age-group (50+ years) (49.4 (95%CI: 28.7-70.1)) (Table 1). Age- and sex-adjusted prevalence of lifetime epilepsy was 23.5/1,000 (95% CI: 11.0-36.0).

Ninety-six (96) reported at least one unprovoked seizure in the 12 months preceding the survey, a crude prevalence of 21.6/1,000 (95% CI: 17.3-25.9) for active epilepsy. The prevalence of active epilepsy was 21.8/1,000 (95% CI: 16.00-27.7) in females and was 21.3/1,000 (95% CI: 15.1-27.6) in males, being similar between the sexes (P=0.898). The prevalence of active epilepsy did not vary significantly by age-group (P=0.260), although it was lowest in the young adults (19-28 year) age-group (14.7 per 1,000 (95%CI: 5.2-24.3)) and highest in oldest age-group (50+ years) (32.9 (95%CI: 15.9-50.9)) (Table 1). Age- and sex-adjusted prevalence of active epilepsy was 16.0/1,000 (95% CI: 5.6-326.4).

### 3.3 Seizure semiology

Of the 141 people with epilepsy, seizure semiology could be accurately determined in 83/141 (58.9%). Convulsive seizures were detected in 59% and non-convulsive seizures in 42% people with epilepsy. Both convulsive and non-convulsive seizures occurred in 7% PWE, only convulsive seizures in 52% and only non-convulsive seizures in 35% PWE (Table 2). Distribution of specific seizures types defining convulsive or non-convulsive seizures are shown in Table 2. Seizures were unspecified in 6%, in whom it was difficult to determine whether they were convulsive or non-convulsive (Table 2). Given the proportion of non-convulsive seizures only of 35%, the minimum prevalence of lifetime epilepsy with non-convulsive seizures was 11.1/1,000 persons (95% CI: 9.31-12.92) based on crude estimates and was 8.2/1,000 persons (95%CI:3.9-12.6) when based on adjusted estimates. Focal seizures comprised 68% of all epilepsies.

### 3.4 Mortality associated with epilepsies with convulsive or non-convulsive seizures

Of the 4,441 people in whom epilepsy status was determined, 146 died (Table 3), which is an annual mortality rate of 2.9 per 1,000 population (95%CI: 2.4-3.4). Of the 146 deaths, 9/96 (9.4%) occurred in those with active epilepsy, being greater than in those without epilepsy (137/4,344 (3.2%); X^2^ P=0.001), and similar with those with inactive epilepsy (1/45 (2.2%); Fisher’s exact P=0.169). The mortality rate in those with lifetime epilepsy was 6.4/1,000/year (95%CI: 3.41-11.80), while it was 2.8/1,000/year (2.3-3.2) in those without epilepsy; hazard ratio (HR) =2.31 (1.22-4.39; p=0.011).

The mortality rate in active epilepsy appeared greater than in inactive epilepsy (Table 3), but the difference did not reach significance levels (HR=4.41 (95%CI: 0.56-34.85; p=0.159)). The mortality rate was (11.2/1,000/year (95%CI: 5.3-23.4)) in those with epilepsy with convulsive and non-convulsive seizures, while none died in those with epilepsy with non-convulsive seizures only (Table 3) (Fisher’s exact P=0.134).

## 4 Discussion

This is the first study in sub-Saharan African to provide an estimate of the prevalence of lifetime and active epilepsy prevalence of both epilepsy with convulsive seizures and non-convulsive seizures, and epilepsy with non-convulsive seizures only in a rural setting. The overall age and sex adjusted prevalence was 23.5/1,000 (95%CI: 11.0-36.0) and did not differ by sex and age groups, an estimate that is greater than for convulsive epilepsies from similar settings [13]. Active epilepsy was common, with the adjusted prevalence being 16.0/1,000 (95% CI: 5.7-326.4), with no differences between sex and age-groups; active epilepsies are easier to recall in the past one year and are the basis for starting anti-seizure medications in Kenya. Convulsive seizures only were reported by more people (52%), than non-convulsive seizures only were (35%), expectedly because the former seizures are easier to detect. Most of these seizures had a focal onset (68%), but it is possible some of these would evolve into generalized seizures under electroencephalography. Adjusted prevalence of epilepsy with non-convulsive seizures only was estimated as 8.23/1,000 persons; these seizures would easily be confused for mental health symptoms and would go untreated if not identified.

The age-adjusted prevalence of all epilepsies of 23.5/1,000 is three times that of active convulsive epilepsy reported in a study from the same area (7.1/1,000) [13], suggesting estimates based on active convulsive epilepsy only grossly underestimate the true prevalence by at least 70%, consistent with suggestions by other authors [7]. The conservative adjusted prevalence of non-convulsive epilepsies of 8.2/1,000 is two times greater than that reported in developed countries (4/1,000) [22], where there is capacity to identify non-convulsive epilepsies. In our study some seizures could not be specified into either convulsive or non-convulsive seizures due to limited information provided by patients, which may have underestimated the proportion used to estimate prevalence. The presence of seizure susceptibility genes in this population [23] and the increased incidence of risk factors such as central nervous system infections [13] explains the high prevalence of epilepsy in resource poor countries compared to developed countries. There are no lifetime estimates for non-convulsive epilepsies in SSA to compare with since the few epidemiological studies that attempted to include non-convulsive seizures focused on the prevalence of active epilepsy only [7,8], the definitions for active epilepsy in these two studies was for seizures in the last 2 or 5 years, unlike our study where seizures in the last year were considered.

Our active epilepsy estimate (16.0/1,000 (95%CI: 5.7-326.4) are substantially greater than those for other studies from Zambia (12.5/ 1,000) [7] and Kenya (10.2/1,000) [8], despite the other two studies having used a less strict inclusion criteria of 2-5 years for active epilepsy that would allow in more epilepsies. The differences may also be related to the methodology used across the studies. For example, our study employed trained clinicians who may detect more people with non-convulsive seizures than lay fieldworkers would. As expected, our active epilepsy estimates are considerably greater than those of studies that did not include non-convulsive epilepsies e.g. studies from Bolivia (11.1/1,000), and China (4.6/1,000) [24]. Active epilepsy is the main criteria used to prescribe anti-epileptic drugs in Kenya, and therefore those with active non-convulsive seizures can benefit from treatment. Other resource limited settings should invest in the training and identification of non-convulsive seizures, some of which can be confused for mental health symptoms [25].

Focal seizures were more common than generalized seizures, similar to a study of active convulsive epilepsy in sub-Saharan Africa [5]. Focal seizures suggest symptomatic epilepsy with easily identifiable preventable causes [26]. Unfortunately, during the study period EEG could not be performed on most people detected with seizures in the community. Seizures types are useful in choice of anti-seizure medications, and a proportion of patients with non-convulsive seizures may require anti-seizure medications such as ethosuximide or sodium valproate for absence seizures [27]. Some non-convulsive seizures e.g. absences can be exacerbated by first generation anti-seizure medications such as phenobarbital, that are widely used in LMIC [28,29]. The high proportion of non-convulsive seizures may indicate childhood absence epilepsy or temporal lobe epilepsy are common in this area [30]. Temporal lobe epilepsy syndromes may be a consequence of acute seizures, malaria-associated seizures or status epilepticus, which are common in this area [31].

This study shows that mortality rate was significantly greater in epilepsy than in those without, which is similar to previous studies from this area [6]. The mortality rates are however lower than that reported in the Ngugi et al. study even when we considered active epilepsy only. This is likely due to methodological differences in these two studies, whereby follow-up in our study was done within the framework of demographic surveillance system, which is done every four months and may miss some mortality cases. In the Ngugi et al. study, PWE were specifically and frequently followed by epilepsy fieldworkers, which may have increased the reporting of mortality. Secondly, our study followed up for a longer period, which increases the denominator person years of observations. Hazard ratios showed similar mortality for active and inactive epilepsy, suggesting that causes or risk factors of mortality are common to both epilepsy types. Mortality was greater in the epilepsies with convulsive and non-convulsive seizures than with only non-convulsive seizures, probably because the former seizures are associated with causes of death such as drowning and accidents. Although stigma may be less in epilepsy with non-convulsive seizures and non-convulsive seizures than in that with convulsive seizures only, both seizures should be identified to optimise treatment.

These estimates are reliable since the diagnosis of epilepsy was made by experienced epilepsy specialists using standard criteria [5, 21]. Use of clinicians trained in the diagnosis of epilepsy improved identification of epilepsy with non-convulsive seizures [19]. Some participants with epilepsy did not describe their seizures to the level of information that would have allowed classification into either epilepsy with convulsive seizures or that with non-convulsive seizures. Clinical history was used to differentiate psychogenic non-epileptic seizures or syncope from epilepsy since video telemetry and ECG were not performed in the field. However, the classification reported in the study is based on semiology only since we did not perform EEG and neuroimaging due to logistic reasons. Coroner’s autopsy reports were not available to conform causes of death, and cultural reasons in this rural community may have affected reporting of deaths. The passive method of follow-up which may not capture deaths in those who emigrated may have underestimated mortality rates.

## 5 Conclusions

The burden of all epilepsies in this area is higher than that of developed countries and suggests that local active convulsive epilepsy estimates grossly underestimate the overall prevalence of epilepsy. The high prevalence of all epilepsies may be related to the increase in the burden of risk factors for epilepsy in this area. Non-convulsive seizures add to the burden of active convulsive epilepsies that are considered the criterion for starting anti-seizure medications in Kenya and other LMIC settings. Although mortality is greater in epilepsy with convulsive seizures plus non-convulsive seizures than that with non-convulsive seizures only, stigma may be similar in both and should be addressed. Assessment by experienced clinicians is expensive, but is the most objective way of obtaining reliable data on epilepsy with non-convulsive seizures in LMIC. Future studies in similar settings using a common methodology are required to enable comparisons.

## Figures and Tables

**Table 1:** Prevalence (per 1,000 persons) of active and life-time epilepsy in Kilifi, Kenya.

				Life-time epilepsy		Active epilepsy	
Age group	Males:N=2,064	FemalesN=2,377	All: N=4,441	Cases: N=141	Prevalence per 1,000 (95% CI)	Cases: N=96	Prevalence per 1,000 (95% CI)
0-5	439	442	881	19	21.56 (11.95-31.17)	13	14.75 (6.77-22.73)
6-12	521	560	1,081	39	36.07 (24.94-47.21)	26	24.05 (14.90-33.19)
13-18	378	320	698	23	32.95 (19.67-46.22)	17	24.35 12.89-35.81)
19-28	309	302	611	18	29.45 (16.01-42.90)	9	14.73 (5.15-24.30)
29-49	255	490	745	21	28.18 (16.27-40.10)	17	22.81 (12.07-33.56)
+50	162	263	425	21	49.41 (28.72-70.09)	14	32.94 (15.90-49.97)

**Table 2:** Classification of seizure types in people with life-time epilepsy in Kilifi, Kenya.

Seizure type	Cases with seizure semiology: N=83 (%)
Convulsive seizures only	43 (51.8%)
Generalised tonic-clonic seizures	10
Generalised other motor seizures	3
Focal to bilateral tonic-clonic seizures	7
Focal motor seizures	18
Other motor seizures eg myoclonic	5
All convulsive seizures (including cases with convulsive and non-convulsive seizures)	49 (59.0%)
Both convulsive and non-convulsive seizures	6 (7.2%)
Non-convulsive seizures only	29 (34.9%)
Generalized absence seizures	8
Focal impaired awareness seizures	19
Focal sensory seizures	3
All non-convulsive seizures (including cases with non-convulsive and convulsive seizures)	35 (42.1%)
Unspecified seizures	5 (6.0%)
Generalized unspecified seizures	1
Focal unspecified seizures	4

**Table 3:** Mortality rates in those with and without epilepsy.

Condition	PYO	Number of deaths	Mortality rate per 1,000 per year
All participants	50,967	146	2.9 (2.4-3.4)
With epilepsy	1,574	10	6.4 (3.4-11.8)
Without epilepsy	49,392	136	2.8 (2.3-3.3)
Active epilepsy	1054	9	8.5 (4.4-16.4)
Inactive epilepsy	520	1	2.0 (0.27-13.7)
Epilepsy with convulsive seizures plus non-convulsive seizures	626	7	11.1 (5.3-23.4)
Epilepsy with non-convulsive seizures only	384	0	0

PYO=person years of observation
